# Factors Associated with the Abandonment of Tuberculosis Treatment in Brazil: A Systematic Review

**DOI:** 10.1590/0037-8682-0155-2022

**Published:** 2023-01-23

**Authors:** Larissa Araújo de Lucena, Gabriela Bezerra da Silva Dantas, Taynara Vieira Carneiro, Hênio Godeiro Lacerda

**Affiliations:** 1 Universidade Federal do Rio Grande do Norte, Graduação em Medicina, Natal, RN, Brasil.; 2 Universidade Federal do Rio Grande do Norte, Departamento de Infectologia, Natal, RN, Brasil.

**Keywords:** Tuberculosis, Treatment adherence, Primary health care, Brazil

## Abstract

**Background:**

Tuberculosis (TB) is a chronic infectious disease caused by *Mycobacterium tuberculosis*. In Brazil, TB is a public health problem, and the treatment dropout rate contributes to it.

**Methods:**

This systematic review investigated the factors associated with TB treatment dropout in Brazil using the Preferred Reporting Items for Systematic Reviews and Meta-Analyses (PRISMA) method. The databases used were Bireme, Scopus, PubMed, Medline, Pan-American Health Organization (PAHO), and Latin-American and Caribbean Literature on Health Sciences (LILACS). The search was conducted on May 16, 2021. Nine articles were reviewed, and all were published within the last 5 years in English, Spanish, or Portuguese.

**Results:**

The sample sizes in the studies ranged from 148 to 77,212 individuals, and the studies enrolled only adult patients (aged 18-59 years) in Brazil. Evidence suggests that the significant risk factors associated with TB treatment dropout are male sex, black race/ethnicity, age between 19 and 49 years, human immunodeficiency virus (HIV) co-infection, low education (<8 years), use of alcohol and illicit drugs, and unsupervised treatment. This study’s limitations were the small number of articles published on this topic with stronger study designs, use of secondary data sources in most articles, and a moderate to high risk of bias in most papers.

**Conclusions:**

There was a significant association between abandonment of TB treatment and HIV/acquired immunodeficiency syndrome co-infection; socioeconomic factors (low education and homelessness); use of alcohol, tobacco, and illicit drugs; and failure to use directly observed treatment. These results can guide more efficient measures to prevent dropout.

## INTRODUCTION

Tuberculosis (TB) is an infectious disease caused by *Mycobacterium tuberculosis*. It is an air-borne infection , primarily infects the lungs, and can potentially affect the entire organism^1^. TB is a public health problem in Brazil, which is among the 30 countries that account for 87% of the global burden of the disease^2^ ^,^ ^3^. In 2020, 66,819 new cases were reported in Brazil, representing an incidence of 31.6 per 100,000 inhabitants, which is lower than the rate observed in previous years. Given the context of the COVID-19 pandemic, this may be due to the reduction in the number of TB notifications at the three levels of care compared to that in 2019, and may have occurred because of the strain on health systems caused by severe acute respiratory syndrome coronavirus 2 (SARS-CoV-2), as well as social isolation^4^.

In 2019, the mortality rate due to TB in Brazil was 2.2 per 100,000 inhabitants^4^ ^,^ ^5^. One contributor to mortality is the treatment dropout rate. Seventy percent of individuals with untreated smear-positive pulmonary TB die within 10 years of diagnosis^1^ ^,^ ^6^. In addition, the effects of dropouts, such as drug resistance and relapse, can increase mortality rates^7^.

The World Health Organization (WHO) recommends cure rates of ≥85% of cases and <5% rate of dropped treatments^2^ ^,^ ^8^. However, in Brazil, the dropout rate is 12% and the cure rate is 70.1%, which is far below the acceptable values^4^.

Non-adherence to treatment has consequences for patients and their contacts. The individual remains ill and the chain of transmission is unbroken. Furthermore, persistent infection culminates in drug resistance and disease recurrence, which hinders the recovery process and increases the time and cost of treatment^9^ ^,^ ^10^.

It has been estimated that by 2035, the mortality rate due to TB in Brazil will be 1.17 per 100,000 inhabitants, with no change in the dropout rate. If there is a 5% reduction in this rate, the estimated mortality would be 0.94 per 100,000 inhabitants, which would allow the global target to be achieved^5^.

Although non-adherence to drug therapy has a relevant impact on the control of infection and mortality, it is a topic that is rarely discussed in the literature, with few studies and no review of factors associated with this outcome in Brazil. Reduction of treatment abandonment is a first-order imperative to reduce mortality caused by TB^5^. The persistence of high rates of non-adherence in Brazil suggests that factors associated with dropout should be discussed more in academia, science, and public administration to expand the direct observed treatment (DOT) strategy and more incisive approaches in health establishments.

Thus, this study aimed to systematically review the factors associated with the abandonment of tuberculosis treatment in Brazil, and to evaluate which factors are the most impactful in the country.

## METHODS

### Protocol

This systematic review was conducted in line with the guidelines of the systematic literature review protocol of the Preferred Reporting Items for Systematic Reviews and Meta-Analyses (PRISMA)^11^.

### Search strategy

The databases used to search for studies were the Latin American and Caribbean Center for Health Sciences Information (BIREME), Scopus, PubMed Medline Pan-American Health Organization (PAHO), and Latin American and Caribbean Literature on Health Sciences (LILACS). The descriptors used were: “abandonment treatment” AND “tuberculosis” AND “Brazil”.

The search was conducted on May 16, 2021, resulting in a total of 248 papers, including 39 articles in Bireme, 25 in Scopus, 184 in PubMed, and zero in PAHO and LILACS.

### Study selection

According to the inclusion and exclusion criteria, the authors (GBSD, LAL, TVC) independently and blindly screened the titles and abstracts of all collated papers, differences were resolved by consensus, and duplicate articles were excluded.

Original studies on the risk factors and socioeconomic profiles of patients (>18 years of age) who abandoned treatment for pulmonary TB were included.

Initially, studies that were published from 2016 to 2021 were included. Therefore, case-control and cohort studies with open access, or those that were made available through the Journal Portal of the Coordination for the Improvement of Higher Education Personnel (CAPES), were included. The authors considered only studies conducted in Brazil that presented abstracts in Portuguese, English, or Spanish. The writers then analyzed the complete texts to check their eligibility.

All other studies with designs different from those mentioned or those that did not present a relationship with the proposed theme were excluded. Studies that used definitions other than those used by the Ministry of Health, which is the non-attendance of the patient to the health unit for over 30 consecutive days after the expected date of return or, in cases of DOT, 30 days after the last medication intake^4^, were excluded.

### Data Extraction

A table was built using Google Sheets to extract data from the full text (Supplementary Table 1). Three independent reviewers (GBSD, LAL, and TVC) performed data extraction individually. The data were categorized according to title, journal, web address, study location, year of publication, sample size, data source, aim, study design, results, and socioeconomic indicators.

The selected studies were separated according to their type. Individual-level work was classified according to study design (case-control, cohort, and clinical trial). Mendeley was used for reference management.

### Quality assessment

Of the initial 248 articles, only nine studies met the inclusion criteria and were included in the analysis. The publications selected for review were evaluated based on similar quality criteria, considering articles published in peer-reviewed journals. For observational studies, the list proposed by the STROBE statement was used as a reference^12^. An adaptation of the STROBE list was used for ecological studies, considering the adequacy of the models, magnitude, and significance of the association.

A Cochrane Collaboration tool, the Quality in Prognosis Studies (QUIPS) tool, was used to evaluate the risk of bias in prognostic studies^13^. The risks of bias in each study are listed in Supplementary Table 2.

### Ethical considerations

This study is based exclusively on published literature and does not require an ethics statement.

### Data analysis

After reading the papers in full, the following data were extracted: title of the article, year of publication and research, place of research, study design, sample size, population characteristics (sex, age, race, years of study, income, drug use, human immunodeficiency virus [HIV] co-infection), and the factors listed as associated with treatment dropout in each analysis.

## RESULTS

The search strategy applied to the databases, to select studies from January 2016 to May 2021 (last 5 years), resulted in 248 articles comprising 39 in Bireme, 25 in Scopus, 184 in PubMed, and zero in PAHO and LILACS. After reading the abstracts, 26 papers were selected for full reading and the inclusion criteria were applied. Duplicate studies were excluded from the analysis, as were those that only mentioned dropout, without mentioning factors that could corroborate this outcome. Finally, nine articles^14^ ^-^ ^22^, with seven retrospective cohort and two prospective cohort studies, were thoroughly analyzed by the evaluators.

**FIGURE 1:**
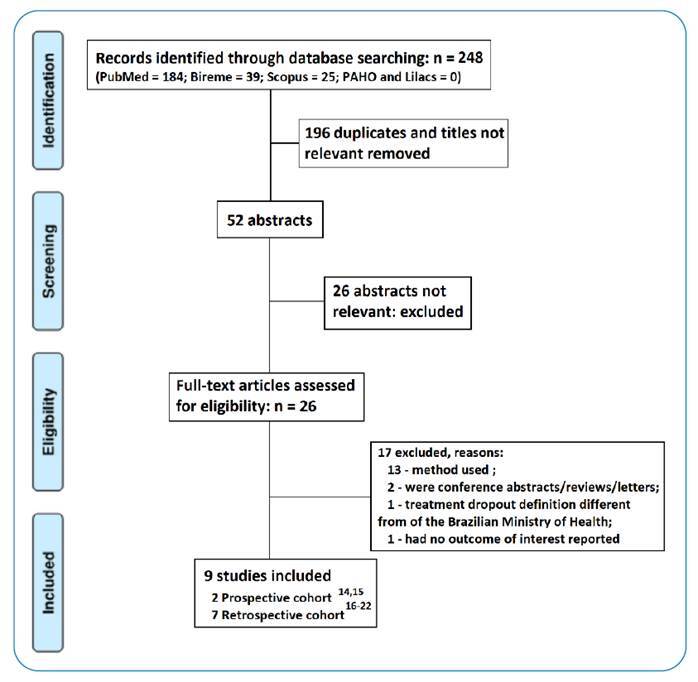
Selection process of articles and results.

Of the 26 articles selected primarily (abstract reading), 13 were discarded because of the methods used (case series, cross-sectional observational, and ecological studies). One study was not selected because the concept of treatment dropout used in that study was different from that of the Ministry of Health^23^. Another study was discarded as it only addressed abandonment without providing evidence of the factors associated with its occurrence^24^. Figure 1 summarizes this process.

The nine selected articles addressed the topic entirely, assessed the profile characteristics of patients who dropped out of tuberculosis treatment in their population for prognostic purposes, described the participants, and computed the losses.

The sample size was measured for the design proposed in each study, with the smallest sample comprising 148 individuals and the largest comprising 77,212 individuals. It is noteworthy that one study was conducted only with people deprived of liberty^15^, whereas another assessed whether housing in slum areas was an associated factor^22^.

The origin and period of data collection were predominantly from secondary sources (SINAN^15^ ^,^ ^18^ ^,^ ^20^ ^-^ ^22^ and TB Web^16^ ^,^ ^17^), during the average period from 2016 to 2021. Two of these studies were carried out with data collected in reference units, one in a secondary reference center in Mato Grosso do Sul^14^ and the other in the INI-FIOCRUZ^16^. As for the geographic locations of the studies, three were conducted in the Northeast region (two in Sergipe^20^ ^,^ ^21^ and one in Paraíba^15^), four in the Southeast region (two in the state of São Paulo^17^ ^,^ ^19^ and two in the state of Rio de Janeiro^16^ ^,^ ^22^), one in the South region (Santa Catarina^18^), and one in the Midwest region (Mato Grosso do Sul^14^).

Regarding the characteristics of the individuals, the most common age group was 19-49 years, with a predominance of male patients in all the studies. Most patients had <8 years of education; a monthly income of three times the minimum wages or less; co-infection with HIV as the main comorbidity; and history of alcohol, tobacco, and illicit drug use.

Notably, the type of treatment used differed between the studies. Some studies compared patients under DOT^18^ with patients who self-administered medications, while another study involved patients with multidrug-resistant TB (MDR-TB)^17^.

The analysis identified the following risk factors associated with abandonment of tuberculosis treatment: sex, race/ethnicity, age, HIV co-infection, low education (<8 years), concomitant use of alcohol and illicit drugs, and unsupervised treatment.

Studies have shown that the use of legal and illegal drugs is significantly associated with lack of treatment adherence. In one study, smokers were reported to be more likely to quit TB treatment than nonsmokers after the analysis of confounding factors (adjusted odds ratio [aOR] = 4.91; 95% confidence interval [CI], 1.08-22.32; p = 0.04)^14^. Non-injectable drugs were associated with a 3-fold higher risk of treatment dropout (OR = 3; 95% CI, 1,308-6,884)^16^, and the use of illicit drugs doubled the chances of dropout (OR = 2.56; 95% CI, 1.02-6.12)^17^. One author compared the dropout rate in the city of Florianópolis/SC (treatment in Basic Health Unit - BHU) with that in the city of São José/SC (specialized unit) and showed that the only sociodemographic indicators that were associated with a significant difference between these populations was the use of illicit drugs (p = 0.02) and homelessness (p = 0.03), and the probability of treatment dropout was 2.41 times higher in Florianópolis (95%; CI, 1.21-4.78) than in São José^18^.

A study that analyzed the impact of alcohol and illicit drug use on tuberculosis treatment outcomes, revealed an association between negative outcomes (death and loss to follow-up) and the use of alcohol (relative risk [RR] = 4.3%; 95% CI, 3.8-4.8), illicit drugs (RR = 5.8%; 95% CI, 5.5-6.1), and both (RR = 5.7%; 95% CI, 5.4-6), with p <0.001^19^. However, this study did not separately show the data on these unfavorable outcomes (death or loss to follow-up). In another study, regarding the risk of treatment dropout, alcohol use was associated with an approximately 2.7-fold increase (OR = 2.728; 95% CI, 2.188-3.402; p <0.001); smoking a 2-fold increase (OR = 1.941; 95% CI, 1.548-2.435; p <0.001); and the use of illicit drugs a 4-fold increase (OR = 3.937; 95% CI, 3.094-5.009; p <0.001) in bivariate analysis^20^. In an analysis of factors associated with dropout in Sergipe, the risk of treatment dropout associated with alcoholism was 21.5%, and that of smoking was 17% (p <0.05)^21^.

Several studies have reported that HIV/acquired immunodeficiency syndrome (AIDS) was associated with abandonment of treatment/treatment dropout. A study involving populations deprived of liberty revealed that patients with AIDS had an approximately 2-fold higher risk of treatment dropout (RR = 1.998; 95% CI, 1.078-3.704; p = 0.028)^14^. Other authors have shown a 3.84-fold higher risk of non-compliance in patients with AIDS (OR = 3.864; 95% CI, 2.604-5.734; p <0.001)^20^. In another study, patients with HIV/TB co-infection had a 22.7% dropout rate^21^.

Among the social aspects highlighted in studies investigating TB, its treatment, incidence, prevalence, and associated challenges, a low education level was associated with treatment dropout in several studies. A study at INI-FIOCRUZ showed that a low level of education represented a 2.5 times greater risk of non-adherence to therapy (OR 2.589; 95% CI, 1.152-5.821)^16^. Homelessness was associated with treatment dropout in two studies, one of which found a significant relationship with an approximately 3-fold risk of an unfavorable outcome (RR = 3.14; 95% CI, 3-3.29; p <0.05)^19^. Incomplete primary education was a characteristic of 14.9% of patients who abandoned treatment in a study carried out in Sergipe (p <0.05)^21^. Another study carried out in Rio de Janeiro/RJ that compared patients who lived in slums and those who did not live in slums, showed a higher risk of DOT abandonment in patients living in slum areas (RR = 1.33; 95% CI, 1.05-1.67; standard error 0.16)^22^.

Three studies addressed DOT within the context of treatment dropout as a factor that influences this outcome. In a cohort study conducted in São Paulo/SP from 2011 to 2015, 25.2% of patients who were not undergoing DOT had adverse outcomes, whereas only 13.1% of those undergoing DOT had adverse events^19^. However, no statistically significant associations were observed between these variables.

A study in Rio de Janeiro/RJ, reported that in a controlled analysis of sex, age, clinical severity of the disease, and HIV/AIDS, DOT was not correlated with a statistically significant reduction in treatment dropout (RR = 1.15; 95% CI, 0.92-1.43). However, patients with TB who lived in slums and were covered under DOT had 0.61 times the risk of dropout compared to patients who lived outside these areas and were not under DOT coverage (95% CI, 0.41-0.90; p <0.01)^22^. In Sergipe, patients under DOT had a lower dropout rate: 9% in the group with DOT and 12.4% in the group without DOT; it was not possible to collect these data in 17.4% of cases (p <0.05)^20^ ^,^ ^21^.

One study listed the type of tuberculosis as a variable that was significantly associated with treatment dropout (pulmonary and extrapulmonary forms with 26.3% dropout and p value = 0.026)^16^. Other factors found only in isolated studies were adverse drug reactions (OR = 6.3; 95% CI, 1.809-21.948)^16^, retreatment (aOR = 3.66; 95% CI, 1.04-12, 88; p = 0.01)^16^, failure to undergo a follow-up bacilloscopy (RR = 5.211; 95% CI, 2.158-12.583; p <0.001)^17^, and mental disorders (36.82%; OR = 2.691; 95% CI, 1.55-4.656; p <0.001)^20^. In another study, previous MDR-TB treatment was listed as a protective factor (OR = 0.33; 95% CI, 0.16-0.66)^17^. In two other studies, it was found that male sex was a contributing element to non-adherence, being associated with a risk of 1.574 (OR = 1.574; 95% CI, 1.232-2.011; p <0.001)^20^ and with a treatment dropout rate of 14.3%^21^.

In a cohort study carried out in a reference unit in Mato Grosso do Sul^16^, a scale was used to grade the risks of treatment dropout in the low, intermediate, and high groups. The variables used were age (over or under 30 years), sex, average income (less than or above the minimum wage), profession, religion, use of illicit drugs, smoking status, and prior treatment. It was possible to establish a significant statistical association only for patients with high-risk scores (OR = 3.43; 95% CI, 0.67-17.77; p = 0.122 for medium; OR = 8.3; 95% CI, 1.65-41.76; p <0.003 for high risk)^16^.

The QUIPS tool was used to analyze the risk of bias in each study, and a detailed analysis of the criteria is provided in Appendix 1. Among the nine studies listed, two were classified as having a high risk of bias, four as moderate, and three as low.

To control the risk of bias, three of the nine evaluated articles employed a multivariate analysis^14^ ^,^ ^15^ ^,^ ^17^, four studies used the Pearson chi-squared test^18^ ^,^ ^19^ ^,^ ^21^ ^,^ ^22^, and two studies used the Poisson regression model^18^ ^,^ ^19^. However, two studies did not specify the tools used to reduce the risk of bias^16^ ^,^ ^20^.

## DISCUSSION

To the best of our knowledge, this is the first systematic review on this topic in Brazil. Few studies have directly analyzed the factors associated with TB treatment dropout in this country, especially with methodologies of greater statistical significance. Among the studies analyzed, the main factors associated with treatment dropout in Brazil were co-infection with HIV/AIDS^14^ ^,^ ^20^ ^,^ ^21^; socioeconomic aspects^16^ ^,^ ^19^ ^,^ ^21^ ^,^ ^23^ (low education, i.e., <7 years of schooling and homelessness); use of alcohol, tobacco, and illicit drugs^14^ ^,^ ^16^ ^,^ ^17^ ^,^ ^18^; and failure to use DOT^22^.

Socioeconomic issues are a crucial epidemiological aspect of the population affected by TB^25^. One effect of poor condition is treatment dropout^25^, with low education and homelessness being elements associated with non-adherence to therapy in this review^16^ ^,^ ^19^ ^,^ ^23^. Safe housing, access to food, employment status, and other factors are external factors that determine health outcomes^26^. When this is not guaranteed, access to healthcare is precarious. Additionally, uncertain routines and financial difficulties contribute to treatment abandonment^26^ ^-^ ^27^.

Another determinant is low education level, which often indicates socioeconomic vulnerability. This characteristic contributes^27^ to the difficulty in understanding and perceiving the disease and its therapeutic approach. Thus, it is essential for health professionals to maintain good communication with patients, especially by adapting the patient’s language, to promote understanding and adherence to treatment^28^.

A strong association was found between TB and HIV coinfection, corroborating other studies’ findings that claim that this association makes treatment adherence difficult^14^ ^-^ ^16^ ^,^ ^18^ ^,^ ^19^ ^,^ ^29^ ^-^ _^31^_. Besides being related to the virus itself, the infection increases the susceptibility to TB^33^, which is closely linked to polypharmacy associated with both treatments^32^ ^-^ ^34^ and the significant number of adverse effects that occur because of drug interactions^35^.

Furthermore, the use of cigarettes, alcohol, and illegal drugs, is also related to a greater propensity for treatment dropout^14^ ^,^ ^16^ ^-^ ^18^ ^,^ ^20^. Smoking had a significant association (p = 0.040^14^, p <0.001^20^), with smokers being more likely to drop out compared to non-smokers^14^ ^,^ ^20^, regardless of the concomitant use of alcohol or drugs^36^. This finding could be because the prevalence of smoking was higher among the lower socioeconomic status groups, which is a risk factor for treatment abandonment, cigarette dependence, and related diseases^14^ ^,^ ^20^ ^,^ ^36^.

Alcohol abuse was related to a higher readmission rate for treatment, which is a predictor of poor adherence and contributes to treatment abandonment^20^ ^,^ ^37^. The critical problems are alcohol-drug interactions and alcohol-related harm events^37^.

Dependence on illicit drugs, regardless of the drug used, was associated with alcoholism in almost all cases, suggesting an overlap between the two variables, both of which play a significant role in treatment abandonment^17^ ^,^ ^19^ ^,^ ^20^. This combination proved even more concerning in MDR-TB cases, with literature showing that approximately half of illicit drug users on their first treatment for MDR-TB abandoned it, and a quarter died^4^. 

These substances can influence TB outcomes through their biological effects and social stigma, and by hindering family support^19^. Thus, illegal drug use, treatment obstacles, marginalization, adverse effects, greater propensity, low adherence to treatment, and limited access represent the most significant challenges in treating drug addicts^14^ ^,^ ^16^ ^-^ ^20^.

Regarding the use of DOT, one study showed a lower dropout rate in the group under DOT (reduction of 3.4%) compared to the group not under DOT^21^. However, two studies did not identify a statistically significant association^19^ ^,^ ^22^.

The Ministry of Health considers DOT to be a minimum of three weekly observations by a healthcare professional on the patient’s medication intake during the whole treatment, totaling at least 24 doses in the intensive phase and 28 doses in the maintenance phase (under a standardized six-month treatment)^21^ ^,^ ^38^.

Thus, corroborated by the findings of other studies, our research also concludes that DOT is crucial for linking individuals to service and care^21^ ^,^ ^29^ ^,^ ^37^ ^,^ ^38^. During DOT, it is possible to diagnose the potential difficulties faced by patients, allowing for intervention when necessary^21^, thereby avoiding treatment dropout. DOT is also associated with an increase in favorable outcomes^38^.

Other contributors to treatment abandonment that were mentioned only in isolated studies, but were statistically significant, included TB type, retreatment, adverse drug reactions^16^, mental disorders, and a lack of follow-up bacilloscopy^20^. Another element associated with this outcome, mentioned in two articles, was the male sex^20^ ^,^ ^21^.

This review has limitations, such as the small number of articles published on this topic, especially those with strong study designs. The population samples used in most articles came from secondary data sources, that could be influenced by factors, such as inadequate filling, data loss, and under-reporting. According to the authors’ analysis using the QUIPS tool, most studies had a moderate to high risk of bias, which limited the generalizability and application of the study’s results. 

## CONCLUSION

In this review, HIV/AIDS co-infection, socioeconomic factors (low education and homelessness), alcohol, tobacco, illicit drugs, and non-use of DOT were significantly associated with TB treatment dropout. Therefore, the application of DOT appears to be a promising strategy to diminish dropout in Brazil, as improvement of socioeconomic factors is more dependent on governmental measures. 

The data should be interpreted cautiously because no meta-analysis or assessment of external validity or heterogeneity was conducted. However, such information can guide more effective measures to prevent dropouts in Brazil among patients presenting with the characteristics discussed. This review reveals the need for more studies on non-adherence to drug therapy with more robust methodologies to achieve lower dropout rates, and meet the global goals established by the WHO. Studies on non-adherence to drug therapy with robust methodologies will not promote lower dropout rates, but can properly describe risk factors and provide policymakers with insight to identify more appropriate strategies to be pursued at the local level.
