# Supplementary material for: Factors Associated with the Abandonment of Tuberculosis Treatment in Brazil: A Systematic Review
Source: Rev Soc Bras Med Trop. 2023 Jan 23;56:e0155-2022. doi: 10.1590/0037-8682-0155-2022 (PMC9870282; doi:10.1590/0037-8682-0155-2022)
Supplement: Supplementary file 1 [file 1678-9849-rsbmt-56-e0155-2022-supp1.pdf]

SUPPLEMENTARY TABLE 1: Data extraction results and characteristics of the analyzed studies.

Authors, Year [Reference]	Title of the Study	Study Design	Sample Size and Characteristics	Factors associated with abandonment
Bezerra *et. al*, 2020 [14]	**Risk stratification and factors associated with TB treatment dropout in a secondary referral unit.**	Prospective cohort	The population of 148 patients (205 patients recruited and 57 excluded) comprised 65% men with a mean age of 43.3 ± 14.76 years, and 58.1% had a laboratory-confirmed diagnosis. Regarding education, 48.8% completed elementary school, and 12% completed high school. Illicit drug use was present in 18.2%. There were 37.2% HIV positive patients, 49.1% had extrapulmonary TB, 16.4% had mixed TB, and 56.8% had isolated pulmonary TB. A total of 23 of the 148 patients underwent retreatment because of dropout or relapse, and 26.1% of these patients dropped out of treatment again.	Retreatment (p = 0.010) and type of TB (p = 0.026) were related to dropout. In the multivariate analysis, smoking and retreatment for TB also had significant associations. After adjusting for potential confounders, smokers had a higher chance of quitting (aOR = 4.91; 95% CI: 1.08, 22.32; p = 0.040) than non-smokers. Patients in retreatment had a greater chance of dropping out than those who were not (aOR = 3.66; 95% CI: 1.04, 12.88). Patients with a high dropout risk score were more likely to interrupt treatment (OR = 8.30; 95% CI: 1.65; 41.76; p = 0.003).
Alves *et. al*, 2020 [15]	**Factors associated with the cure and abandonment of tuberculosis treatment in the population deprived of liberty.**	Prospective cohort	Data from SINAN (Paraíba), from January 2007 to December 2016, showed 614 notifications of new cases arising from the PPL, with 93.8% (n = 576) being males.	There was a statistically significant association between the outcomes (cure or abandonment) and AIDS (p = 0.044), HIV (p = 0.048), and not having a follow-up bacilloscopy (p = 0.001). The proportion of dropouts was significantly higher among individuals with AIDS (33.3%) and those who did not undergo follow-up bacilloscopy (21.2%). AIDS (relative risk - RR = 1.998; 95% confidence interval - 95% CI 1.078 - 3.704; p = 0.028) and not having follow-up bacilloscopy (RR = 5.211; 95% CI 2.158 - 12.583; p < 0.001) remained significantly associated with the dropout outcome. Dropout rates at >29 years and below were similar (16.8% and 17%, respectively).
Cardoso *et. al*, 2017 [16]	**Outcomes of tuberculosis treatment and factors associated with each of them in a cohort followed between 2010 and 2014.**	Retrospective cohort	A total of 2079 individuals were selected. The mean age was 40.32 (15.19) years, with a slight predominance of men (56%). Most had a monthly income of less than three minimum wages (70.6%). The predominant clinical form of TB was pulmonary, and most participants had attended high school. There were 24 HIV-infected patients, and 57% used antiretroviral drugs during the TB treatment. Culture confirmed most TB cases.	A low level of education (incomplete elementary school) (OR 2.589; CI 1.152–5.821), the use of non-injectable drugs (OR 3; CI 1.308–6.884), and interruption of treatment because of adverse reactions (OR 6.3; CI 1.809–21.948). The logistic model showed good discrimination with a ROCAUC of 0.81 and a 2 of 0.29.
Arroyo *et. al*, 2019 [17]	**Predictive model of unfavorable outcomes for MDR-TB**	Retrospective cohort	Regarding all cases diagnosed with MDR-TB between 2006 and 2015 in the state of São Paulo on the notification system (TBWeb), the study data was composed of 802 patients of MDR-TB (after filtering the duplicate notifications). Most were white (44.6%), followed by black/brown (33.3%), aged between 30-59 years (66.3%), of which 70% were male, and 44.1% had less than seven years of education. About 13% were diagnosed with AIDS and 13% with DM. About 22.8% reported alcoholism, and 12% used illicit drugs. Of all participants, 76% were undergoing supervised treatment.	One or more previous treatments for MDR-TB (ORaj = 0.33; 95% CI 0.16–0.66) was a protective factor for dropout, while the use of illicit drugs was a risk factor (ORaj = 2.56; 95% CI 1.02-6.12). The number of previous treatments was a protective factor for death (ORaj = 0.41; 95% CI 0.18–0.90), while the associated risk factors were diagnosis in emergency services or during hospitalization hospital (ORaj = 2.88; 95% CI 1.28–6.33) and use of illicit drugs (ORaj = 2.06; 95% CI 1.36–5.59).
Scheffer *et. al*, 2018 [18]	**Performance of centralized versus decentralized tuberculosis treatment services in Southern Brazil, 2006-2015**	Retrospective cohort	Evaluated patients were treated for pulmonary TB in the cities of Florianópolis and São José, using two data sources, each analyzing a different period. Data from the ten years (2006 to 2015) were gotten from the SINAN, available on the TABNET platform of the DIVE / SC, and a prospective cohort study was carried out with patients with culture-confirmed pulmonary TB who started treatment between 2014 and 2015. Of the 2309 cases evaluated (2006-2015), 207 patients were interviewed from 2014 to 2015 in Florianópolis and São José-SC. The majority in both cities were men (66.9 and 63.8, respectively), with less than seven years of education (71.8 and 60.2), aged 25-34 years (32.3%) for Florianópolis, and > 45 years (34.9) for São José. In both cities, over 60% were smokers; 45.9% used illicit drugs in Florianópolis while in São José, this percentage did not reach 30%.	Regarding sociodemographic indicators, the only significant differences observed between the two populations were the proportion of illicit drug users (p = 0.02) and the proportion of homeless patients (p = 0.03), which were predominant indicators in Florianopolis. For patients who started treatment for TB in Florianópolis, where the UBS provided care, the treatment success rate was 22% lower (95% confidence interval = 9–33%), and the probability of treatment dropout was 2.41 times higher (95% confidence interval = 1.21–4.78) when compared to patients who started treatment in São José, where care was provided in the outpatient clinic.<br>The TB treatment dropout rate (45%, 95% CI 12–90%) was also higher in the municipality with decentralized care. During the two-year follow-up, significant differences were found between patients in centralized and decentralized care regarding treatment success (84.5 vs. 66.1%), treatment dropout (10.7 vs. 25.8%), use of illicit drugs (27.7 vs. 45.9%), and homelessness (3.6 vs. 12.9%).
Pelissari and Diaz-Quijano, 2018 [19]	**Impact of alcohol disorder and illicit drug use on tuberculosis treatment results: a retrospective cohort study**	Retrospective cohort of patients diagnosed with TB from 2011 to 2015 in the state of São Paulo, Brazil.	The study counted 77,212 patients with TB. Among them, 22,498 were female, while 54,714 were male. The total number of participants aged between 15 and 34 was 36,080, between 34 and 49 was 21,681, and 50 years or more was 19,451. Regarding race, 35,707 were non-black, while 33.135 were black. Regarding the presence or absence of HIV, 7,239 were seropositive compared to 69.973 who were seronegative. Sixty-two thousand two hundred seventy-one had a diagnosis of pulmonary TB, while 11,903 had an extrapulmonary form of the disease. As for the other variables analyzed, most were not in prison (68,228), had a home (68.228), and did DOT (54,940).	The incidence of unsuccessful TB treatment outcomes was as follows: 17% had alcohol and drug use disorders, 13.8% were female, and 18.6% were male. As for age, the highest prevalence of poor outcomes in the treatment of TB occurred in patients aged 50 years or more (19.9%). Among patients aged 34-49 years, this result corresponded to 18.8%. The prevalence was higher among black people (18%), those living with HIV (37.5%), those with pulmonary forms of TB (17.4%), non-detainees (18.5%), homeless people (51.3%), and those without DOT (25.2%).
Lima *et. al*, 2021 [20]	**Early identification of individuals at risk of losing TB treatment follow-up: a generalized hierarchical analysis**	Retrospective cohort of patients diagnosed with TB in Sergipe / Brazil from 2015 to 2018	Of the 2,449 patients with TB analyzed, 1,749 were male. The largest racial group was brown individuals (1,654); most individuals were aged between 20-39 (1,304). Regarding years of education, the group with the highest education (729 individuals) was between 4-7 years. Other variables analyzed were the clinical form of TB (2175 with pulmonary form); diagnosis of AIDS (present in 111); alcohol use (present in 567); DM (present in 183); mental disorder (57); drug use (379); smoking (551); population deprived of liberty (280); homelessness (74); immigrants (10); recipients of government benefits (190).	Loss to follow-up represented 18.21%, which was prevalent among men (20.03%), blacks (20.34%) and people with brown skin color (19.55%), people aged 20-39 years (21.87%), and people with a few years of education. Individuals who lived in urban areas (19.64%), those who restarted treatment after loss to follow-up (58.13%), and those with pulmonary TB (19.08%) had a higher dropout rate. People with AIDS (42.33%), mental illness (36.82%), alcohol use (31.05%), drug use (39.39%), or smoking (26.54%) had higher negligence than individuals without these features. Bivariate logistic regression revealed that people who were male, dark-skinned, aged between 20 and 39 years old, had no or only a few years of education, were urban dwellers or homeless, had pulmonary TB, reentered after treatment dropout, had AIDS or a mental disorder, used alcohol or illicit drugs, and smoked were more likely to drop out.
Santos *et. al*, 2019 [21]	**Factors associated with death and treatment dropout in new cases of tuberculosis in Sergipe, Brazil**	Longitudinal study, historical Cohort type, from the new TB cases identified among 2007 and 2016.	This study analyzed SINAN data. A total of 5989 new TB cases were registered, of which 95.4% were included. Among the total, 68.2% were male, and the most prevalent groups with respect to age corresponded to the groups from 20 to 29 years (25.9%) and 30 to 39 years (21.9%). Regarding race, 64.5% were brown. Regarding education, 46,6% had not completed elementary school, compared to 0.7% who had completed higher education. 94.1% made up part of the population not deprived of liberty. The health region with the highest number of participants was Aracajú (46.3%).	The dropout rate was higher in men (14.3%), in the age groups from 20 to 39 years old (17.6%), and in those with incomplete primary school education (14.9%). DOT was performed in 52% of cases, with lower dropout rates (9.0%) and death (6.0%). HIV/TB co-infection was identified in 4.9% of cases, of which 22.7% dropped out and 31.7% died.
Snyder *et. al*, 2016 [22]	**Risk factors for differential outcome following directly observed treatment (DOT) of slum and non-slum tuberculosis patients: a retrospective cohort study**	Retrospective cohort analytics of TB cases registered in SINAN.	Of 6601 TB patients, 2.317 (35.1%) were women. The mean age was 38.7 years (standard deviation: 16.5). One thousand eight hundred and seven patients lived in slums, 638 (35.2%) were under DOT, while 1,234 (26.2%) patients who lived in slum areas were not under DOT.	After controlling for sex, age, severity of disease, and HIV/AIDS, it was found that TB patients who lived in slums were 1.33 times more likely to drop out of treatment (95% CI: 1.05-1.67) than patients with TB who did not live in slums. However, TB patients living in slum areas and with DOT had 0.61 times the risk of non-compliance (95% CI: 0 .41-0.90) compared to TB patients not in AGSN and not under DOT.

Legend of the illustration	
TB	Tuberculosis
MDR-TB	Multidrug-resistant Tuberculosis
DOT	Directly Observed Treatment
TBWeb	Tuberculosis Patient Control System
SINAN	System of Information on Notifiable Diseases
TABNET	Health Information
DIVE / SC	Department of Epidemiological Surveillance of Santa Catarina
HIV	Human Immunodeficiency Virus
PPL	Persons Deprived of Liberty
AIDS	Acquired Immunodeficiency Syndrome
DM	Diabetes Mellitus
SC	Santa Catarina
OR	Odds Ratio
CI	Confidence Interval
RR	Relative Risk
ROC/AUC	Receiver Operating Characteristic/Area Under the Curve
UBS	Basic Health Unit
AGSN	Subnormal Clusters
