# Supplementary material for: Factors Associated with the Abandonment of Tuberculosis Treatment in Brazil: A Systematic Review
Source: Rev Soc Bras Med Trop. 2023 Jan 23;56:e0155-2022. doi: 10.1590/0037-8682-0155-2022 (PMC9870282; doi:10.1590/0037-8682-0155-2022)
Supplement: Supplementary file 2 [file 1678-9849-rsbmt-56-e0155-2022-supp2.pdf]

SUPPLEMENTARY TABLE 2: Analysis of the risk of bias in each study.

	RISK OF BIAS	RISK OF BIAS ASSESSMENT								
		Bezerra *et. al* [14]	Alves *et. al* [15]	Cardoso *et. al* [16]	Arroyo *et. al* [17]	Scheffer *et. al* [18]	Pelissari and Diaz-Quijano [19]	Lima *et. al* [20]	Santos *et. al* [21]	Snyder *et. al* [22]
	**Study participation**									
	The source population or population of interest is adequatel described for key characteristics	OK	OK	OK	OK	OK	OK	OK	OK	OK
	The sampling frame and recruitment are adequately described, possibly including methods to identify the sample, place of recruitment, and period of recruitment	OK	OK	OK	OK	OK	OK	OK	OK	OK
	Inclusion and exclusion criteria are adequately described	OK	OK	OK	OK	OK	OK	OK	OK	x
	There is adequate participation in the study by eligible individuals	OK	OK	OK	OK	OK	OK	OK	OK	OK
**RESUME**	**The study sample represents the population of interest on key characteristics, sufficient to limit potential bias of the observed relationship between the prognostic factor and outcome**	LOW	LOW	LOW	LOW	LOW	LOW	LOW	LOW	MODERATE
	**Study Attrition**									
	Response rate is adequate and is > 80%.	X	OK	OK	OK	OK	OK	OK	OK	OK
	Attempts to collect information on participants who dropped out of the study are described.	OK	OK	OK	OK	OK	X	X	X	X
	Reasons for loss to follow up are described.	OK	OK	OK	OK	OK	OK	OK	OK	OK
	There are no important differences between key characteristics and outcomes in participants who completed the study and those	OK	OK	OK	OK	OK	X	X	X	OK
**RESUME**	**Loss to follow-up is not associated with key characteristics sufficient to limit potential bias to the observed relationship between the prognostic factor and the outcome.**	MODERATE	LOW	LOW	LOW	LOW	MODERATE	MODERATE	MODERATE	MODERATE
	**Prognostic Factor Measurement**									
	A clear definition or description of the prognostic factors is provided.	X	OK	X	OK	OK	OK	X	OK	OK
	Method of prognostic factor measurement is adequately valid and reliable to limit misclassification bias.	OK	OK	OK	OK	OK	OK	OK	OK	OK
	The method and setting of measurement of PF is the same for all study participants.	OK	OK	OK	OK	OK	OK	OK	OK	OK
	More than 80% of the study sample has completed data for PF variable.	OK	OK	OK	OK	OK	OK	OK	OK	X
**RESUME**	**PF is adequately measured in study participants to sufficiently limit potential bias**	MODERATE	LOW	MODERATE	LOW	LOW	LOW	MODERATE	LOW	MODERATE
	**Outcome Measurement**									
	A clear definition of the Outcome is provided.	OK	OK	OK	OK	OK	OK	OK	OK	OK
	The method of outcome measurement used in valid and reliable to limit misclassification bias.	OK	OK	OK	OK	OK	OK	OK	OK	x
	The method and setting of outcome measurement is the same for all study participants.	OK	OK	OK	OK	OK	OK	OK	OK	OK
**RESUME**	**Outcome of interest is adequately measured in study participants to sufficiently limit potential bias**	LOW	LOW	LOW	LOW	LOW	LOW	LOW	LOW	MODERATE
	**Study Confounding**									
	All important confounders are measured	OK	OK	X	OK	X	OK	OK	OK	OK
	Clear definitions of the important confounders measured are provided.	OK	X	X	OK	X	OK	OK	OK	X
	The method and setting of confounding measurement are the same for all study participants.	OK	OK	X	X	X	OK	OK	OK	OK
	Important potential confounders are accounted for in the study design and the analysis.	OK	OK	X	OK	X	X	X	OK	OK
**RESUME**	**Important potential confounders are appropriately accounted for, limiting potential bias with respect to the relationship between PF and outcome.**	LOW	MODERATE	HIGH	MODERATE	HIGH	MODERATE	MODERATE	LOW	MODERATE
	**Statistical Analysis and Reporting**									
	There is sufficient presentation of data to assess the adequacy of the analysis.	OK	OK	X	OK	OK	OK	OK	OK	OK
	The selected statistical model is adequate for the design of the study.	OK	OK	OK	OK	OK	OK	OK	OK	OK
	There is a description of the association of the prognostic factor and the outcome, including information about the statistical significance.	OK	OK	X	OK	OK	OK	OK	OK	x
	There is no selective reporting of results.	OK	OK	OK	OK	OK	OK	X	OK	OK
**RESUME**	**The statistical analysis is appropriate for the design of the study, limiting potential for presentation of invalid or spurious results.**	LOW	LOW	MODERATE	LOW	LOW	LOW	MODERATE	LOW	MODERATE
	RISK OF BIAS	MODERATE	LOW	MODERATE	LOW	MODERATE	MODERATE	HIGH	LOW	HIGH

Risk classification	Risk of each session
Low	All criteria were met.
Moderate	≤ 2 criteria not met.
High	≥ 3 criteria not met.
	Risk of each item
Low	All low or even moderate.
Moderate	Two or three domains rated moderate or one rated high.
High	≥ 2 rated as high or > 3 as moderate.

Analysis of the risk of bias of each study.

Legend of the illustra	PF = Prognostic Factor
